# Subphenotype mapping in systemic lupus erythematosus identifies multiple novel loci associated with circulating interferon alpha

**DOI:** 10.1186/ar4626

**Published:** 2014-09-18

**Authors:** Silvia N Kariuki, Yogita Ghodke-Puranik, Jessica M Dorschner, Beverly S Chrabot, Jennifer A Kelly, Betty P Tsao, Robert P Kimberly, Marta E Alarcón-Riquelme, Chaim O Jacob, Lindsey A Criswell, Kathy L Sivils, Carl D Langefeld, John B Harley, Andrew D Skol, Timothy B Niewold

**Affiliations:** 1 University of Chicago, Chicago, IL, USA; 2 Mayo Clinic, Rochester, MN, USA; 3 Gwen Knapp Center for Lupus Research, University of Chicago, Chicago, IL, USA; 4 Oklahoma Medical Research Foundation, Oklahoma City, OK, USA; 5 University of California, Los Angeles, CA, USA; 6 University of Alabama, Birmingham, AL, USA; 7 GENYO Centre for Genomics and Oncological Research: Pfizer/University of Granada/Andalusian Regional Government, Grenada, Spain; 8 University of Southern California, Los Angeles, CA, USA; 9 Rosalind Russell/Ephraim P. Engleman Rheumatology Research Center, University of California, San Francisco, CA, USA; 10 Wake Forest University, Winston-Salem, NC, USA; 11 Cincinnati Children's Hospital Medical Center and Cincinnati VA Medical Center, Cincinnati, OH, USA, USA

## Background

Systemic lupus erythematosus (SLE) is a chronic autoimmune disorder characterized by inflammation of multiple organ systems, loss of tolerance to self-antigens, and dysregulated interferon responses. SLE is both genetically and phenotypically heterogeneous, and we hypothesize that this greatly reduces the power of overall case-control studies in SLE. Increased circulating level of the cytokine interferon alpha (IFNα) is a stable, heritable trait in SLE which has been implicated in primary disease pathogenesis. Forty to 50% of patients have high IFNα, and high levels correspond with clinical differences.

## Methods

To study genetic heterogeneity in SLE, we performed a case-case study comparing patients with high versus low IFNα in over 1,800 SLE cases. Four hundred European ancestry cases formed the discovery GWAS set, and 1,443 cases from a large independent multi-ancestral replication cohort were used to validate associations.

## Results

In meta-analysis, the top associations in European ancestry were PRKG1 rs7897633 (*P*_Meta _= 2.75 × 10^-8^) and PNP rs1049564 (P_Meta _= 1.24 × 10^-7^). We also found evidence for cross-ancestral background associations with the *ANKRD44 *and *PLEKHF2 *loci. These loci have not been previously identified in case-control SLE genetics studies. Bioinformatic analyses implicate these loci functionally in dendritic cells and natural killer cells, both of which are involved in IFNα production in SLE.

## Conclusions

As case-control studies of complex heterogeneous diseases reach a limit of feasibility with respect to subject number and detectable effect size, the study of informative pathogenic subphenotypes becomes a highly attractive and efficient strategy for genetic discovery in complex human disease.

